# Influence of the Manufacturing Method on the Adhesion of *Candida albicans* and *Streptococcus mutans* to Oral Splint Resins

**DOI:** 10.3390/polym13101534

**Published:** 2021-05-11

**Authors:** Andrea Schubert, Ralf Bürgers, Franziska Baum, Oliver Kurbad, Torsten Wassmann

**Affiliations:** Department of Prosthodontics, University Medical Center Goettingen, Robert-Koch-Str. 40, 37075 Goettingen, Germany; ralf.buergers@med.uni-goettingen.de (R.B.); franziska.baum@stud.uni-goettingen.de (F.B.); oliver.kurbad@med.uni-goettingen.de (O.K.); torsten.wassmann@med.uni-goettingen.de (T.W.)

**Keywords:** oral splint, CAD/CAM, 3D printing, milling, digital dentistry, *Candida albicans*, *Streptococcus mutans*, microbial adhesion

## Abstract

Microbial adhesion to oral splints may lead to oral diseases such as candidiasis, periodontitis or caries. The present in vitro study aimed to assess the effect of novel computer-aided design/computer-aided manufacturing (CAD/CAM) and conventional manufacturing on *Candida albicans* and *Streptococcus mutans* adhesion to oral splint resins. Standardized specimens of four 3D-printed, two milled, one thermoformed and one pressed splint resin were assessed for surface roughness by widefield confocal microscopy and for surface free energy by contact angle measurements. Specimens were incubated with *C. albicans* or *S. mutans* for two hours; a luminometric ATP assay was performed for the quantification of fungal and bacterial adhesion. Both one-way ANOVA with Tukey post hoc testing and Pearson correlation analysis were performed (*p* < 0.05) in order to relate manufacturing methods, surface roughness and surface free energy to microbial adhesion. Three-dimensional printing and milling were associated with increased adhesion of *C. albicans* compared to conventional thermoforming and pressing, while the *S. mutans* adhesion was not affected. Surface roughness and surface free energy showed no significant correlation with microbial adhesion. Increased fungal adhesion to oral splints manufactured by 3D printing or milling may be relevant for medically compromised patients with an enhanced risk for developing candidiasis.

## 1. Introduction

Oral splints are used in several fields of dental practice, including the treatment of temporomandibular disorders [1,2], the protection of teeth from excessive occlusal forces arising from parafunctional behaviors such as bruxism [3] and orthodontic tooth alignment [4].

Conventional oral splint manufacturing is performed by the vacuum thermoforming or pressing of cold- or heat-cured acrylic resins, or by a combination of both. Recently, computer-aided design and computer-aided manufacturing (CAD/CAM) have updated oral splint manufacturing [5]. Fully digital workflows involve intraoral scanning of dental arches, software-supported splint design and computer-aided splint manufacturing by additive or subtractive procedures [6,7,8]. Additive manufacturing by three-dimensional (3D) printing technologies is based on the light-induced polymerization of liquid resin monomers, forming solid objects layer by layer. Subtractive procedures are performed by computerized numerical control (CNC) devices milling objects from pre-fabricated, highly-polymerized resin blanks [9]. CAD/CAM manufacturing shows high accuracy, standardization and reproducibility, and is both time- and cost-efficient [6,9,10]. This has led to a continuous replacement of conventional splint manufacturing [6,11]. The mechanical properties of 3D-printed and milled oral splints have been found to be satisfactory for clinical use [5,12]. However, surprisingly little is known about their biological properties.

It is a well-known fact that, shortly after application in the oral cavity, the hard surfaces of the biomaterials are colonized by microorganisms that form structured communities called biofilms [13,14]. Among the over 750 species of the oral microbiome, there are some with outstanding clinical significance due to their role as pathogens that cause gingivitis, periodontitis, caries or candidiasis [15]. The opportunistic yeast *Candida albicans* has been shown to colonize numerous dental materials, including those on a resin base [16,17,18]. While it is mostly harmless to healthy individuals, it may cause oral and systemic candidiasis in immunocompromised hosts, such as patients suffering from AIDS or undergoing chemotherapy or radiation as part of cancer therapy [19,20,21]. Furthermore, patients wearing complete dentures, and especially those with poor oral hygiene, are likely to develop *Candida*-associated denture stomatitis [22]. Similar to complete dentures, oral splints are made of acrylic resins and are worn for many hours daily, in direct contact with the oral mucosa. Therefore, oral splints should be considered as a reservoir for *C. albicans* infections that impose adverse oral or systemic effects, especially in medically or orally compromised patients.

*Streptococcus mutans* is a Gram-positive oral bacterium that constitutes an important etiologic agent in dental caries, which is the most prevalent oral disease in general [23,24]. *S. mutans* metabolizes carbohydrates into lactic acid, which then demineralizes tooth surfaces, causing carious lesions [25]. Beyond its role in cariogenicity, *S. mutans* is pivotal for the creation of a low-pH milieu for other acidogenic and aciduric microorganisms, which ultimately results in the formation of complex pathogenic biofilms [26]. Systemically, the presence of *S. mutans* is linked to severe medical conditions such as bacterial endocarditis and atherosclerosis [23]. Due to the multiple metabolic implications of *S. mutans*, it is clinically significant to assess its adhesion to oral splint resin materials that cover large areas of tooth surfaces.

The adhesion of microorganisms to biomedical surfaces is influenced by material composition, surface roughness and surface free energy [27,28,29,30]. Furthermore, surface topography, such as irregularities or porosities, has been theorized to influence a material’s susceptibility to the adherence of the microorganisms [31,32,33,34]. Some features of surface topography are determined by the respective manufacturing method; milled resin materials show lower porosity than conventional cold- or heat-cured resin materials [31]. In 3D-printed resin materials, on the other hand, microscopic indentations can be found at the interface of the layers formed during the manufacturing process [35]. To the best of our knowledge, there are no studies assessing the influence of the manufacturing method of oral splint resins on microbial adhesion.

The aim of the present in vitro study was to investigate the initial adhesion of *C. albicans* and *S. mutans* to 3D-printed, milled and conventionally manufactured oral splint resins, and to correlate these findings with both the surface characteristics and the manufacturing method.

## 2. Materials and Methods

### 2.1. Specimen Preparation

Cylindrical specimens with a diameter of 10 mm and a height of 2.5 mm were manufactured from four 3D-printed, two milled and two conventional (one thermoformed, one pressed) splint resins (Table 1). In brief, rods of each resin were produced according to the manufacturers’ instructions and sliced into disks using a separating machine (Micracut 201, Metkon, Bursa, Turkey). Surface treatment of the specimens was performed with an automated grinding machine (Digiprep 251, Metkon, Bursa, Turkey) and silicon carbide grinding paper with descending abrasiveness.

### 2.2. Surface Roughness

The arithmetical mean roughness values (Ra) were calculated for five specimens of each tested resin at three sites via widefield confocal microscopy (Zeiss Smartproof 5, Carl Zeiss, Jena, Germany) and automated software analysis (ConfoMap ST 7.4.8076, Carl Zeiss, Jena, Germany).

### 2.3. Surface Free Energy

For the determination of surface free energy, contact angle measurements were performed: 1 µL of distilled water and 1 µL of methylene iodide were applied to the specimen’s surface. Within 30 s after application, a computer-aided measurement device (Drop Shape Analyzer DSA25, Krüss, Hamburg, Germany) performed ten contact angle measurements for each liquid. The surface free energy was calculated using the formula introduced by Owens and Wendt [36].

### 2.4. Microbial Culture

*C. albicans* (lot no. 1386, Deutsche Sammlung von Mikroorganismen und Zellkulturen, DSMZ, Braunschweig, Germany) and *S. mutans* (lot no. 20523, DSMZ) were cultured under standard conditions in Universal Medium for Yeasts (lot no. 186, DSMZ) or Trypticase Soy Yeast Extract Medium (lot no. 92, DSMZ). Both microorganisms were harvested by centrifugation, washed twice with phosphate-buffered saline (PBS, Merck, Darmstadt, Germany) and resuspended in PBS. The suspensions of *C. albicans* or *S. mutans* in PBS were adjusted to an optical density of 0.3 at 600 nm by densitometry (Bio Photometer, Eppendorf, Hamburg, Germany) [37].

### 2.5. Luminescence Assay

Under sterile conditions, resin specimens were transferred to 24-well plates and attached to well bottoms using silicone (Z-Dupe, Henry Schein Dental, Langen, Germany). Then, 1 mL of *C. albicans* or *S. mutans* suspension was added to each well and incubated for 2 h at 37 °C and 55 rpm. The viable cells were quantified using an adenosine triphosphate (ATP)-based luminescence assay (LT07-221, Lonza, Cologne, Germany). After washing with PBS twice in order to remove non-adherent cells, 300 µL of a cell lysis reagent was added to each well in order to extract ATP. After 10 min, 100 µL of the supernatant was transferred to a 96-well plate, where 100 µL of ATP monitoring reagent plus was added to each well. After 5 min of incubation, luminescence was measured using a plate reader (FLUOstar Omega, BMG Labtech, Ortenberg, Germany) at a preset gain of 4000. Standard glass specimens (Paul Marienfeld, Lauda-Koenigshofen, Germany) served as controls.

### 2.6. Microbial Staining

Hoechst staining was performed exemplarily for each resin after microbial incubation. In brief, specimens were washed three times with 0.85% saline. Then, 1 mL bisbenzimide H 33342 trihydrochloride (Sigma Aldrich, Munich, Germany) was added to each specimen for 13 min. Staining solution was removed via three washing steps with 0.85% saline, and microbial cultures were fixated using 8% paraformaldehyde solution. After 10 min, specimens were dried and mounted on object slides for visualization via fluorescence microscopy (BZ-X710, Keyence, Osaka, Japan).

### 2.7. Statistical Analysis

Statistical analyses were performed using GraphPad Prism 9 (GraphPad Software, San Diego, CA, USA). The overall level for significance was set at *α* = 0.05.

For the analysis of surface roughness and surface free energy, means and standard deviations were calculated. After Q-Q plotting for normal distribution and Levene’s testing for homogeneity of variance, one-way ANOVA and Tukey’s multiple comparison post hoc analysis were applied.

Data from luminescence assays are shown as medians with box-and-whisker plots. For the analysis of microbial adhesion, data were tested for normal distribution (Q-Q plotting) and variance homogeneity (Levene’s test). One-way ANOVA for the factors “oral splint resin” and “manufacturing method”, and Tukey’s multiple comparison post hoc analysis, were performed. Pearson correlation analysis was used to determine the correlation between Ra and microbial adhesion, or surface free energy and microbial adhesion, respectively.

## 3. Results

### 3.1. Surface Characteristics

The resin specimens were mechanically polished until high-gloss surfaces were obtained. Confocal microscopy showed no correlation between surface irregularities and the specific manufacturing processes. Ra means ranged from 0.038 (M-PM crystal, standard deviation: 0.007) to 0.092 µm (FREEPRINT ortho 385, SD: 0.008). Surface free energy means varied from 62.67 (±3.43, Erkodur) to 70.86 mN/m (±0.31, Dental LT Clear). Statistical analysis indicated significant differences between the resins for both the Ra and surface free energy (Table 2).

### 3.2. Microbial Adhesion

**Figure 1:**
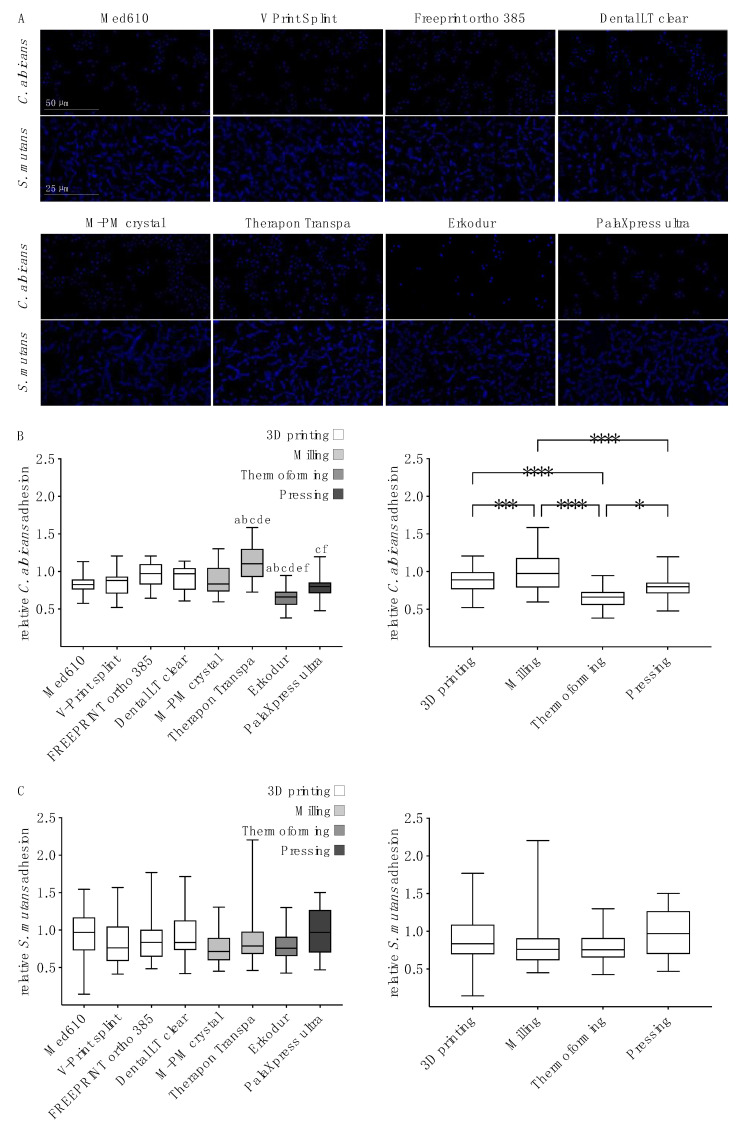
Microbial adhesion to oral splint resins. (**A**) Hoechst staining (blue fluorescence) exemplarily visualizes the adhesion of *C. albicans* (**upper** rows) and *S. mutans* (**lower** rows) to the test materials (columns). *C. albicans* yeast cells form small heterogenous clusters; *S. mutans* cells are arranged in typical chain formations. (**B**), relative *C. albicans* adhesion according to the luminescence assay. *C. albicans* adhesion shows significant differences between test materials (**left**). ^a^ *p* < 0.05 compared with Med610, ^b^ *p* < 0.05 compared with V-Print splint, ^c^ *p* < 0.05 compared with FREEPRINT ortho 385, ^d^ *p* < 0.001 compared with Dental LT clear, ^e^ *p* < 0.001 compared with M-PM crystal, ^f^ *p* < 0.0001 compared with Therapon Transpa. Arrangement of the data according to the underlying manufacturing methods (**right**) also shows significant differences for *C. albicans* adhesion. * *p* < 0.05, *** *p* < 0.001, **** *p* < 0.0001. Glass was used for normalization (=1.0). (**C**), relative *S. mutans* adhesion according to the luminescence assay. There were no significant differences in *S. mutans* adhesion between the test materials (**left**) or between the manufacturing methods (**right**). Glass was used for normalization (=1.0).

Hoechst staining was performed in order to visualize the adhesion of *C. albicans* and *S. mutans* to the resins (Figure 1A). *C. albicans* cells that were present in the ovoid yeast formed as small clusters with a heterogeneous surface distribution. *S. mutans* was organized in typical chains that were scattered over the surfaces.

Microbial adhesion was quantified according to the relative luminescence of an ATP-based assay. The *C. albicans* adhesion (Figure 1B, left) to Therapon Transpa (milled) was significantly higher than to any other resin. There were no significant differences between the 3D-printed resins. The thermoformed resin Erkodur showed a significantly lower susceptibility to the adherence of *C. albicans* than the 3D-printed and milled resins. In order to investigate the influence of the manufacturing method on microbial adhesion, resins were grouped according to the applied manufacturing methods (Figure 1B, right). Milling resulted in significantly more fungal accumulation than 3D printing, thermoforming or pressing. Three-dimensional printing and pressing were associated with significantly higher *C. albicans* adhesion than thermoforming.

For *S. mutans* adhesion, there were no significant differences between the resins (Figure 1C, left) or between the manufacturing methods (Figure 1C, right).

Pearson’s correlation analysis showed a slightly positive but statistically insignificant correlation between the surface roughness and adhesion of both *C. albicans* (correlation coefficient = 0.388, *p* = 0.343) and *S. mutans* (correlation coefficient = 0.354, *p* = 0.390). There was no correlation between the surface free energy and adhesion of *C. albicans* (correlation coefficient: 0.158, *p* = 0.709). A slightly positive correlation between the surface free energy and adhesion of *S. mutans* was revealed, but it was statistically insignificant (correlation coefficient: 0.433, *p* = 0.284).

## 4. Discussion

The present study investigated the adhesion of *C. albicans* and *S. mutans* to 3D-printed, milled, thermoformed and pressed oral splint resins for the first time. Microbial adhesion was assessed in order to correlate fungal and bacterial colonization with surface characteristics and manufacturing methods. To ensure that only viable cells were subjected to the relative quantification of biofilm accumulation, a well-established, reproducible and highly sensitive ATP-based luminescence assay was performed [16,37,38,39].

The influence of surface roughness on microbial adhesion to dental materials has been investigated extensively and, in general, rough surfaces have been correlated with higher microbial accumulation than smooth surfaces [28,40,41]. Quirynen et al. reported that Ra values below 0.2 µm did not further reduce the biofilm accumulation on titanium surfaces in vivo [40]. This specific value has often been cited as a threshold for the “smoothness” of dental materials, below which no further biofilm reduction can be achieved [17,28,42]. Conversely, it has been shown that even very low Ra values of 30 nm and below affect bacterial adhesion to biomaterials [43]. Our findings indicated a slightly positive but insignificant correlation between surface roughness and microbial adhesion below the Ra threshold of 0.2 µm (*p* = 0.343 for *C. albicans*, *p* = 0.390 for *S. mutans*). We concluded that surface roughness did not significantly affect the adhesion of *C. albicans* and *S. mutans*. Details about the role of surface roughness on microbial adhesion to biomaterials should be clarified by further research.

There is evidence to suggest that surface topography may exert a greater influence on microbial adhesion than surface roughness [33]. However, we did not find topographic characteristics, such as porosities or indentations, to be attributed to a specific manufacturing method. Hence, the substantial effects of surface topography on microbial adhesion must be denied within the limitations of the present experimental setting.

Surface free energy has been shown to influence the adhesion of various cell types to dental materials [16,44,45]. Modifying surface free energy can have promoting or inhibiting effects on microbial adhesion, depending on the experimental conditions [46,47,48]. In an investigation on denture base and lining materials, an increase in surface free energy resulted in an increase in *C. albicans* adhesion [49]. For the adhesion of *S. mutans* to composite resins, evidence is conflicting as both insignificant and significant correlations between surface free energy and adhesion have been reported [50,51]. In the present study, surface free energy was not significantly correlated with microbial adhesion, although for *S. mutans*, a slightly positive (but insignificant) correlation was observed.

*C. albicans* adhesion varied significantly between resins and manufacturing methods. While, in previous studies on denture base resins, milling decreased *C. albicans* adhesion compared to conventional manufacturing [32,52], and 3D printing increased fungal adhesion [52], our findings indicated an increase in fungal adhesion to both milled and 3D-printed splint resins. Remarkably, within the group of milled resins, Therapon Transpa was significantly more susceptible to fungal adhesion than the M-PM crystal. This finding suggests that the specific physicochemical properties of the two resins exerted a greater influence on adhesion than the manufacturing method itself. Although all of the assessed resins were based on polymethyl methacrylate, individual material composition may vary in terms of the type of initiator and additive, as well as the content of residual monomers, which may affect the biological properties [53,54,55,56,57]. Material composition and monomer elusion were not within the scope of the present study; they need detailed analysis in future investigations in order to clarify our rather speculative interpretation.

It is noteworthy that several 3D printing technologies relevant to clinical dentistry were included in the present investigation, namely poly jet modelling (Med610), digital light processing (V-Print Splint, Freeprint ortho 385) and stereolithography (Dental LT clear) [11,58]. No significant differences in *C. albicans* and *S. mutans* adhesion were found between these technologies, suggesting that the printing technology was not decisive for microbial adhesion.

Evidence on *S. mutans* adhesion to polymeric dental materials in vitro is conflicting, with both minor [59,60] and major differences [61,62] reported between materials. However, the comparability of these studies is limited, due to the different experimental settings adapted to the focus of each research question. In the present study, the adhesion of *S. mutans* showed little difference between the tested resins. Hence, the mechanisms involved in the initial attachment of *S. mutans* were widely unaffected by the physicochemical variations in the tested resins, while *C. albicans* reacted more sensitively to them. In line with this, Ozel et al. reported different susceptibilities of resin-based provisional crown materials to the adherence of *C. albicans* and *S. mutans* [62]. The role of species-specific characteristics, such as the cell surface protein antigen SpaP of *S. mutans* [63,64] or the adhesion protein Hyphal wall protein 1 of C. albicans [65,66], remains to be clarified in the context of the initial attachment to dental resins. Differences in the ATP metabolism of fungi and bacteria might additionally explain the differential results for the two microorganisms in the performed ATP-based luminescence assay of the present study [67,68,69].

Some limitations must be noted in interpreting the results of the present study. We performed an in vitro study, which implies the benefits of standardized conditions at the expense of differing from the actual environmental conditions in the oral cavity. The adhesion of *C. albicans* and *S. mutans* was assessed alone, while the oral microbiome consists of a multitude of species that interact in complex biofilms [14,15]. However, we chose *C. albicans* and *S. mutans* as representatives of oral biofilms with high pathogenic potential. Although oral candidiasis can be caused by several fungal species, *C. albicans* is the predominant causative agent [70]. Likewise, there are numerous bacterial species associated with dental caries, such as *lactobacilli* [71,72]. However, the outstanding role of *S. mutans* in the initial phase of caries etiology is indisputable [71], and the associated high clinical relevance justifies the assessment of its adhesion in the present study.

There is evidence to suggest that *C. albicans*-secreted polysaccharides promote *S. mutans* adhesion both in vitro and in vivo [73]. It would be of interest to investigate this synergism in future dual-species biofilms on dental polymers.

Under in vivo conditions, an acquired pellicle deriving from saliva substrates influences microbial adhesion to hard surfaces [74]. There are inconsistent data on whether the pellicle enhances or inhibits the adhesion of *C. albicans* to dental resins [75,76]. In an in vitro study on *S. mutans* adhesion to composite resins, the presence of saliva had no decisive influence on bacterial adhesion [33]. As our experimental design was carefully chosen to investigate the influence of material characteristics of dental splint resins, we excluded additional parameters to avoid further complicating the interpretation of results.

## 5. Conclusions

Within the limitations of the present study, we conclude that the novel CAD/CAM technologies, 3D printing and milling, increase the susceptibility of oral splint resins to the adherence of *C. albicans*, compared to conventional manufacturing. Our findings might be clinically relevant for medically and orally compromised patients with a likelihood of developing oral or systemic candidiasis and, therefore, need validation by further in vitro and in vivo investigations.

## Figures and Tables

**Table 1:** Specification of the splint resins investigated in this study.

Manufacturing Method	Product	Manufacturer
3D printing	Med 610	Stratasys, Eden Prairie, MN, USA
	V-Print splint	Voco, Cuxhaven, Germany
	FREEPRINT ortho 385	Detax, Ettlingen, G
	Dental LT Clear	Formlabs, Somerville, MA, USA
Milling	M-PM crystal	Merz Dental, Luetjenburg, Germany
	Therapon Transpa	Zirkonzahn, Gais, Italy
Thermoforming	Erkodur	Erkodent, Pfalzgrafenweiler, Germany
Pressing	PalaXpress ultra	Kulzer, Hanau, Germany

**Table 2:** Surface characteristics after surface treatment. Data are expressed as means and standard deviations; SFE = surface free energy.

Manufacturing Method	Resin	Ra (µm)	SFE (mN/m)
3D printing	Med610	0.074 ± 0.013 ^d,f,g^	69.61 ± 1.30 ^d–g^
	V-Print Splint	0.077 ± 0.009 ^d–g^	68.44 ± 1.98 ^e,f^
	FREEPRINT ortho 385	0.091 ± 0.008 ^a,b,d–g^	69.81 ± 2.16 ^d–g^
	Dental LT clear	0.064 ± 0.014 ^a–c,f,g^	70.86 ± 0.31 ^d–g^
Milling	M-PM crystal	0.038 ± 0.007	65.31 ± 0.88
	Therapon Transpa	0.064 ± 0.010 ^d,f,g^	63.66 ± 3.09
Thermoforming	Erkodur	0.043 ± 0.010 ^f^	62.67 ± 3.43
Pressing	PalaXpress ultra	0.046 ± 0.006 ^g^	65.02 ± 2.41

^a^*p* < 0.05 compared with Med 610, ^b^ *p* < 0.01 compared with V-Print splint, ^c^ *p* < 0.0001 compared with FREEPRINT ortho 385, ^d^ *p* < 0.05 compared with M-PM crystal, ^e^ *p* < 0.05 compared with Therapon Transpa, ^f^ *p* < 0.01 compared with Erkodur, ^g^ *p* < 0.05 compared with PalaXpress ultra.

## Data Availability

The data presented in this study are openly available in the data repository ‘Göttingen Research Online’ (https://doi.org/10.25625/RQSCAB).
